# Genetic influences on diet in young Swedish adults: a twin study

**DOI:** 10.1016/j.ajcnut.2026.101199

**Published:** 2026-01-13

**Authors:** Lisa Kastenbom, Simon Haworth, Linda Eriksson, Ralf Kuja-Halkola, Ingegerd Johansson, Anders Esberg

**Affiliations:** 1 Department of Odontology, Umeå University, Umeå, Sweden; 2 Bristol Dental School, University of Bristol, Bristol, United Kingdom; 3 Department of Medical Epidemiology and Biostatistics, Karolinska Institute, Stockholm, Sweden

**Keywords:** diet, dietary patterns, nutrients, heritability, genetic, twin study

## Abstract

**Background:**

Dietary choices are shaped by both genetic predisposition and environmental exposures, yet the relative influence of these factors remains insufficiently understood across populations and age groups. Young adulthood represents a critical period when long-term eating habits take form, and clarifying the determinants of dietary behavior in this life stage may inform strategies to promote sustained health.

**Objectives:**

This twin study aimed to estimate genetic and environmental contributions to food, energy, and nutrient intakes, and taste preferences in young adults in Sweden.

**Methods:**

The study included 2832 Swedish twins (858 monozygotic and 1974 dizygotic; mean age 24 y; 59.5% female). Participants completed a validated dietary questionnaire assessing food intake frequencies and taste preferences. Additive genetic (A), shared environmental (C), and nonshared environmental (E) influences on a priori dietary indices, specific food and nutrient intakes, and taste preferences were estimated using classical ACE twin models and nested models fitted in OpenMx.

**Results:**

Heritability estimates across dietary traits ranged from 20% to 61%. Genetic influences on overall dietary pattern indices exceeded 40%. Heritability varied across food groups (e.g., 61% for venison; 24% for potatoes) and nutrient intakes (50% for fiber; 20% for sodium), indicating differing degrees of genetic impact across dietary components. Taste preferences also showed substantial genetic contributions (21%–61%), with the strongest effects observed for bitter foods (e.g., black coffee, grapefruit), followed by sweet foods (e.g., jam/marmalade).

**Conclusions:**

This large-scale twin study provides a comprehensive overview of genetic and environmental influences on dietary behavior in young adults, showing substantial genetic and nonshared environmental contributions across diverse dietary traits. These results provide a foundation for future research on diet–disease relationships and may support the development of prevention and intervention strategies, including emerging precision-nutrition approaches.

## Introduction

Dietary intake is essential for developing and maintaining health and avoiding disease. According to the WHO, a healthy diet prevents both malnutrition and overnutrition, with reduced risk of poor health outcomes, often manifesting as noncommunicable diseases [1]. A growing body of research demonstrates that diets rich in fruits, vegetables, whole grains, fish, and unsaturated fats, typical of Mediterranean or plant-forward patterns, are associated with reduced cardiovascular disease risk [2], whereas Western dietary patterns high in saturated fat, processed meat, and added sugars increase risk. Similar associations have been observed for inflammatory and autoimmune conditions, including rheumatoid arthritis, where plant-based diets may alleviate symptoms and high intake of processed foods may exacerbate them [3]. Respiratory conditions, such as asthma and chronic obstructive pulmonary disease, also appear more favorable among individuals consuming antioxidant-, fiber-, and ω-3 (n–3)-rich diets, in contrast to Western dietary patterns emphasizing refined carbohydrates and processed meats [4]. Diet has additionally been linked to risk of several cancers, with plant-based, high-fiber diets showing protective associations and diets high in red and processed meats, alcohol, and ultraprocessed foods associated with increased incidence [5,6]. Globally, diets low in whole grains, fruits, vegetables, nuts and seeds, and ω-3 fatty acids, but high in sodium, remain leading contributors to lower compared with higher mortality [7].

Human dietary behavior is shaped by both genetic and environmental influences [[8], [9], [10], [11], [12], [13]]. The degree to which genetic differences account for variation in a trait is known as heritability. Reported heritability estimates for specific food items typically range from 20% to >60%, with shared environmental effects generally small or absent and remaining variance explained by nonshared environmental factors or measurement errors [8,12,14,15]. Heritability for dietary behaviors, such as restraint or uncontrolled eating, has been reported at 45%–60% [16], nutrient and energy intakes show estimates of 20%–45% [9,17], food preferences at 20%–60% [10,11,13,18], and broader dietary patterns at 25%–50% [8,15,19,20].

Previous work has mainly focused on limited dietary components or selective populations, making cross-study comparisons difficult (Supplemental Table 1). To address this gap, the present exploratory twin study estimates additive genetic (A), shared environmental (C), and nonshared environmental (E) contributions to a broad set of dietary traits, including a priori dietary indices, food group intakes, nutrient intakes, and taste preferences, among young adult Swedish twins.

## Method

### Study population

The Swedish Twin Registry (STR) is one of the world's largest and most comprehensive twin registries, containing data on >200,000 twins. In 2004, The Child and Adolescent Twin Study in Sweden (CATSS) was initiated, collecting information at 9 or 12, 15, 18, and 24 y of age, with data used for several outcomes. The present study sample was drawn from the 24-y follow-up of the CATSS24. The CATSS24 includes comprehensive dietary data, anthropometrics, and updated lifestyle and health information. Participants were young adults (mean age 24 y) living across Sweden, and the cohort is considered broadly representative of the Swedish population with respect to sociodemographic characteristics. Twins are predominantly of Nordic/European ancestry, reflecting the national demographic composition [[21], [22], [23]].

Information on diet, zygosity, sex, age, birth year, and self-reported height and body weight was compiled for CATTS24 participants who *1*) had answered a food frequency questionnaire (FFQ) in 2021, 2022, or 2023, and *2*) had confirmed zygosity from a DNA-based method [monozygotic (MZ) or dizygotic (DZ) twins]. To reduce the impact of diet-reporting bias, twins with an extreme estimated BMI (kg/m^2^) from self-reported height and weight were excluded. Here, 2 participants with an estimated BMI < 15 and 1 with an estimated BMI > 140 were excluded. In addition, those for whom the dietary recording did not pass quality control (described below). Out of 4181 twins with a dietary recording, 1349 were excluded in the FFQ quality control or being in an incomplete twin pair. This left a final study group of 2832 participants (858 MZ, 1974 DZ) in 1416 complete twin pairs.

### Ethical approval

The “CATSS24” received ethical approval from the Regional Ethical Review Board in Stockholm (Dnr 2015/1947-31/4, date 30 October, 2015) and an addendum for this project was approved by the Swedish Ethical Review Authority (Dnr 2021-04797). For twins enrolled through the STR, informed consent was obtained prior to participation and covers the collection, linkage, and research use of questionnaire data, clinical measures, and registry information. Participation is voluntary and can be withdrawn at any time.

### Dietary assessment

Dietary assessment was based on FFQ data, using a questionnaire developed to capture modern dietary habits in Sweden. It records food intake frequencies for 108 food items on a 9-level scale and has 4 photos of plates with increasing portion sizes for staple foods, meat/fish/vegetarian options, and vegetables. Data captured using the questionnaire have been evaluated against repeated 24-h recalls with acceptable validity and high reproducibility [24].

Reported frequencies for the 108 food/food-aggregate questions in FFQ2020 were transformed to intakes per day (0–4 intakes/d) and aggregated to estimate intakes in 30 food groups (Supplemental Table 2) used per se or to calculate scores for 3 a priori diet pattern indices. These were the relative Mediterranean Diet Index (rMED) with scores between 0 and 18 [25], the Healthy Nordic Food Index (HNFI) with scores between 0 and 6 [26], and the Plant-Based Diet Index (PDI) with scores between 18 and 90 [27]. The 3 food indices were calculated according to original definitions [25,26,27], with the modification that “olive oil” in rMED was calculated from total vegetable oil use, as the FFQ2020 does not distinguish olive oil from other cooking oils (Supplemental Table 3). Daily energy and nutrient intakes were estimated using sex- and age-specific portion sizes for the weighting of nutrient contents given in the database at the Swedish Food Agency, as described in the recent validation study [24]. In addition, taste preferences for sweet, bitter, and sour tastes were assessed by asking participants to rate their liking of 6 representative foods on a 6-level scale ranging from disgust to love. Median imputation was used for missing questions or questions answered with “do not know” or “do not want to answer.”

Participants with dietary recordings with ≥10% missing answers or incomplete portion size indications in the FFQ2020, or an implausible estimated daily energy intake (<500 or >6000 kcal) or physical activity level (<0.3 or >4) were excluded.

### Descriptive statistics

Descriptive statistics of the study population were calculated as percentages and frequencies or means with SD or 95% confidence interval (95% CI), depending on metric scale or variable distribution.

### Heritability analysis, the classical twin model

The classical twin model is a well-established approach for estimating the relative influence of genetic and environmental factors on the variation of a trait. This model relies on a few fundamental assumptions, that is *1*) that MZ and DZ twins experience equal environmental exposures relevant for developing the trait [28], *2*) that MZ twins share essentially 100% of their alleles, whereas DZ twins share on average 50% of their cosegregating alleles, and *3*) that genetic and environmental factors act additively and independently, meaning that there are no gene–environment interactions influencing the trait. Under these assumptions, any greater similarity observed between MZ twins compared with DZ twins can be attributed to genetic effects rather than shared environment. In the present study, the term “genetic effects” follows the conventional twin modeling sense to denote the share of phenotypic variance explained by additive genetic factors, with the understanding that these estimates depend on the assumptions of the ACE model and should not be interpreted as causal effects [29].

Differences in within-pair similarity in MZ and DZ twins, respectively, allow partitioning of the total phenotypic variance into ACE effects. The nonshared environmental factors (E) explain differences between twins from the same family (with in pair) due to factors other than shared genetics and experiences, including nonshared environmental exposures and measurement errors [28]. These estimations can either be done by calculating twin correlations and applying Falconer’s formula to obtain rough estimates of heritability [h2 = 2(rMZ − rDZ)] or by using structural equation modeling (SEM) within an ACE framework. The SEM approach allows simultaneous estimation of the ACE components while accounting for covariates and providing model fit statistics, such as the Akaike Information Criterion (AIC) and likelihood ratio tests [29]. In this study, heritability estimates were derived using the SEM-based ACE model implemented in OpenMx (R v. 4.3.2, R Foundation for Statistical Computing).

### Estimation of genetic effect

The suitability of the data for heritability analyses was assessed by calculating the correlation separately for rMZ and rDZ pairs, and if rMZ > rDZ, this was interpreted as an indication of a possible genetic influence on the phenotype.

All dietary traits were adjusted for relevant covariates using linear regression models, yielding standardized residuals (mean = 0, SD+/1) which were used for twin modeling. Covariates included in each model were selected based on subject-matter knowledge (established associations with dietary intake) with support of statistical significance. For food groups and nutrient variables, adjustments included sex, body weight, total energy intake, and a sex–energy interaction term to control for sex differences in dietary patterns. Total energy intake was adjusted for sex and body weight. Taste preferences were adjusted for sex, and food indices were adjusted for sex, body weight, and total energy intake. Missing body weight values were imputed using sex-specific median weights. Covariates that lacked theoretical justification or were not statistically significant were excluded from the final models.

All dietary variables were checked for skewness, with those exceeding skewness >±1 considered highly skewed and subjected to log_10_ transformation. Distributions were examined posttransformation to confirm an acceptable distribution. Skewed variables were log_10_ transformed prior to standardizing the residuals (mean = 0, SD ±1).

Assumptions for the classical twin model, meaning tests for equal means (Welsh 2 sample *t*-test) and equal variances (*F*-test) were tested. Variables violating the assumptions (Bonferroni correction *P* < 0.05) between MZ and DZ twins were excluded. A data analysis flowchart is presented in Supplemental Figure 1.

**FIGURE 1:**
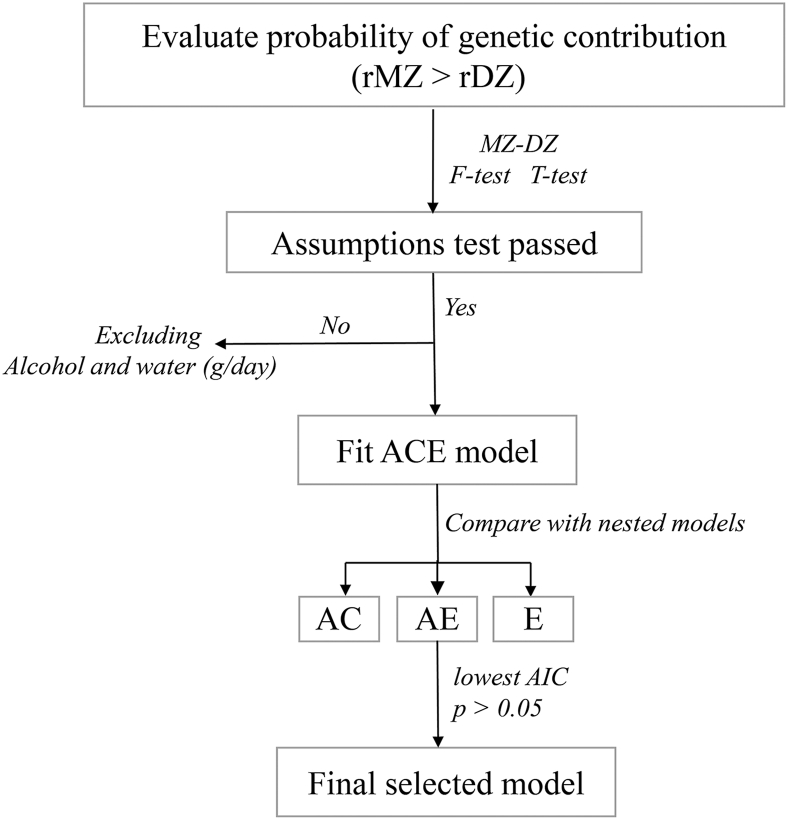
Flowchart describing the model selection procedure. Step 1: calculating twin correlation to evaluate the probability of genetic contribution (rMZ > rDZ). Step 2: test model assumptions and exclude variables that do not pass. Step 3: fit the full ACE model. Step 4: compare ACE model with simpler, nested models (AC, AE, E). Step 5: select the most parsimonious model based on the lowest AIC and nonsignificant *P* values (*P* > 0.05). AIC, Akaike Information Criterion; rDZ, dizygotic; rMZ, monozygotic.

Univariate ACE models were fitted to estimate the components A, C, and E for each dietary variable. The full ACE model was first fitted, and then compared with the nested AE, CE, and E models to identify the most parsimonious model for each trait. The most parsimonious model was selected by comparing nested models (AE, CE, E) and choosing the one that was not significantly worse than the full ACE model while exhibiting the lowest AIC. The process of model selection is presented in Figure 1. Evaluation of the statistical significance of the A was done by comparing the full ACE model with the nested CE model using a likelihood ratio test. A deterioration in model fit when excluding the A component indicates that genetic factors contribute to the variance of the trait. To address multiple comparisons, Benjamini–Hochberg false discovery rate adjustment was used to the ACE compared with CE *P* values across all traits. For each trait, point estimates and 95% CI limits for the A, C, and E components were obtained.

Data processing and extraction of descriptive statistics were carried out using SPSS v29, and heritability analyses were performed using the OpenMx package (penmx.psyc.virgina.edu) in R (v. 4.3.2) (cran.r-project.org).

## Results

### Participant characteristics and reported dietary intake

An overview of the demographics for the 2832 participants (858 MZ, 1974 DZ) in 1416 complete twin pairs is presented in Table 1. The participants were 24 y old, and 59.5% were female with a mean BMI of 24. Overall, 28.5% of the participants were classified as overweight [BMI ≥25] and 4.4% as underweight [BMI <18.5]. The estimated energy intake was on average 2013 kcal/d, and a mean (SD) g/d intake of carbohydrates of 193.4 (84.8), fat 92.0 (44.7), protein 78.6 (43.1), and total sugar (glucose, fructose, and sucrose) 64.4 (31.6). Most participants (88.8%) adhered to an omnivorous diet, with few reported being vegetarians (5.6%), pescatarians (3.7%), or vegans (1.9%), respectively. In addition, 10.4% of participants followed a lactose-free diet, and <3% adhered to a gluten-free, dairy-free, or other specialized diets because of hypersensitivity or intolerance (Table 1).

**TABLE 1:** Overview of characteristics for all participants and stratified by zygosity (MZ or DZ)

Characteristics	All	MZ	DZ
Participants (*n*)	2832	858	1974
Sex, females (%)	59.5	62.7	58
BMI (kg/m^2^), *n* (%)			
<18.5	116 (4.1)	44 (5.2)	72 (3.7)
18.5–24.9	1867 (67.4)	577 (68.3)	1290 (67.0)
25–29.9	593 (21.3)	174 (20.7)	419 (21.5)
≥30	203 (7.2)	50 (5.9)	153 (7.8)
kcal/d, mean (SD)	2013 (870.1)	2003 (850.2)	2016 (878.8)
Diet type [*n* (%)]			
Omnivore	2513 (88.8)	764 (89.0)	1749 (88.7)
Vegetarians	158 (5.6)	52 (6.1)	106 (5.4)
Pescatorian	105 (3.7)	25 (2.9)	80 (4.1)
Vegan	53 (1.9)	17 (2.0)	36 (1.8)
Special diet [*n* (%)]			
Lactose free	295 (10.4)	90 (10.5)	205 (10.4)
Gluten-free	66 (2.3)	20 (2.3)	46 (2.3)
Dairy-free (nonmilk)	44 (1.6)	14 (1.6)	30 (1.5)
Other	76 (2.7)	28 (3.3)	48 (2.4)

Abbreviations: MZ, monozygotic twins; DZ, dizygotic twins.

### Assumption testing and correlation analysis

Prior to fitting the ACE models, similarity within twin pairs was evaluated to assess indications of genetic influence. Higher correlations among MZ compared with DZ twins indicate the presence of additive genetic effects. In addition, assumptions of the classical twin model, meaning equal means and variances across zygosity groups, were tested to ensure that the data met the requirements for subsequent SEM.

The Pearson correlation coefficients between twin 1 and twin 2 in twin pairs were higher among MZ twins (mean 0.40) compared with DZ twins (mean 0.15) for all dietary traits, indicating possible additive genetic influence on all dietary traits (Supplemental Table 4). Assumptions for the classical twin model, meaning tests for equal means (Welsh 2 sample *t*-test) and equal variances (*F*-test), were found acceptable for all variables except for the estimates of alcohol and water (both g/d). Consequently, these variables were excluded from further analysis (Supplemental Table 5). Excluding the A component in the ACE models resulted in a poorer fit of the model for all variables, indicating a significant A on all dietary traits (Supplemental Table 6).

### Genetic driving forces behind dietary patterns

To evaluate if overall dietary patterns carry evidence of genetic influence, adherence to 3 established dietary indices was first summarized. The variance in these indices was then partitioned into genetic and environmental components using classical twin modeling.

**FIGURE 2:**
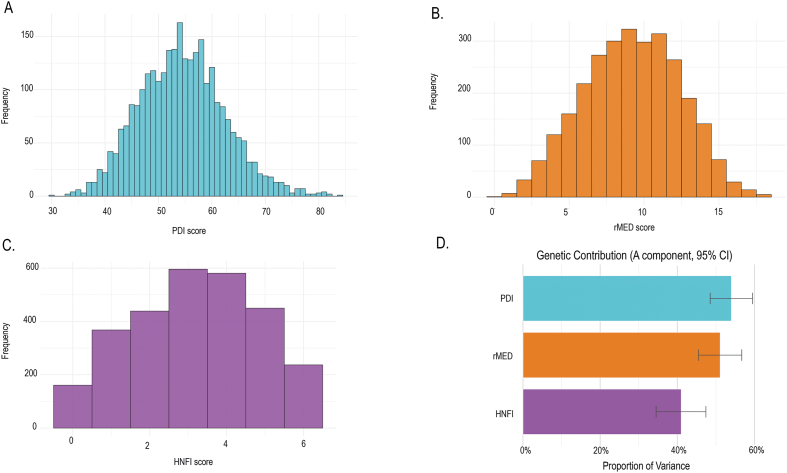
Distribution of dietary indices and genetic contribution to variance. (A–C) Histograms illustrating the distribution of 3 dietary indices: (A) Plant-Based Diet Index (PDI), (B) relative Mediterranean Diet Index (rMED), and (C) Healthy Nordic Food Index (HNFI). (D) Bar plots of the estimated proportion of variance explained by additive genetic effects (heritability) for PDI, rMED, and HNFI, based on ACE model results. Error bars represent 95% confidence intervals (CIs).

Adherence to all 3 dietary patterns was moderate, with a mean (SD) score of 54.4 (8) for PDI, 9.2 (3.2) for rMED, and 3.2 (1.7) for HNFI (Figure 2A–C). The trends were similar if tested together or for the MZ and DZ twins separately. For models estimating the proportion of variance in dietary indices explained by additive genetics, the AE model demonstrated the most parsimonious model characterized by the lowest AIC value (Supplemental Table 6). In these young adult Swedes, >40% of the variance in dietary patterns (PDI, rMED, HNFI) could be attributed to genetic differences. Specifically, the genetic estimates (95% CI) were 54.0% (48.5%, 59.5%) for PDI, 51.1% (45.5%, 56.7%) for rMED, and 41.0% (34.5%, 47.4%) for HNFI (Figure 2D).

### Genetic driving forces behind different foods

**FIGURE 3:**
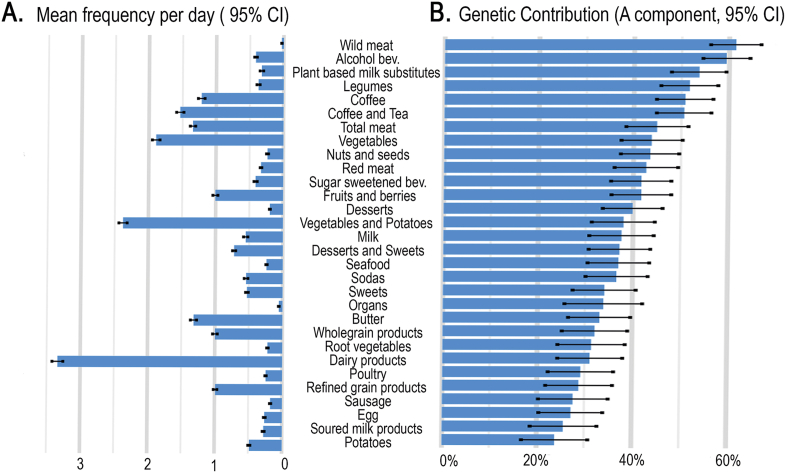
Mean daily intake frequency (A) and estimation of the proportion of variance explained by additive genetic effect (B) of 30 food groups. Error bars indicate the 95% confidence interval (CI).

Variation in intake of food groups was examined, and variance in intakes was then decomposed into genetic and environmental components using classical twin modeling. The intake frequencies of the 30 food groups ranged from 0 to 19 intakes/d (Supplemental Table 7). Intake of dairy products was most frequent, with, on average, 3.3 intakes/d, followed by vegetables and potatoes (2.4 intakes/d), vegetables (1.9 intakes/d), and coffee and tea (1.5 intakes/d). The AE model was the most suitable for assessing the proportion of variance explained by genetics across the 30 food groups. The genetic influence on food consumption varied considerably, with estimates (95% CI) ranging from 61.4% (56.1%, 66.7%) to 23.8% (16.8%, 30.8%). The findings included genetic contribution >50% for consumption of the foods: venison 61.4% (56.1%, 66.7%), alcoholic beverages 59.4% (54.5%, 64.4%), plant-based milk substitutes 53.7% (47.9%, 59.4%), legumes 51.7% (45.7%, 57.7%), coffee and tea 50.6% (44.8%, 56.3%), and coffee 50.8% (44.8%, 56.7%). Just below mean additive genetic influence of 50% was intake of total meat 44.9% (38.4%, 51.5%), vegetables 43.8% (37.4%, 50.3%), nuts and seeds 43.5% (37.3%, 49.6%), red meat 42.7% (36.0%, 49.4%), sugar-sweetened beverages 41.7% (35.3%, 48.0%) fruits and berries 41.7% (35.4%, 48.0%), desserts 39.9% (33.6%, 46.2%). No food group was estimated with a genetic contribution <20% (Figure 3, Supplemental Table 6). These results highlight the varying importance of genetic factors associated with the intake of different food groups.

### Genetic driving forces behind energy and nutrient intake

**FIGURE 4:**
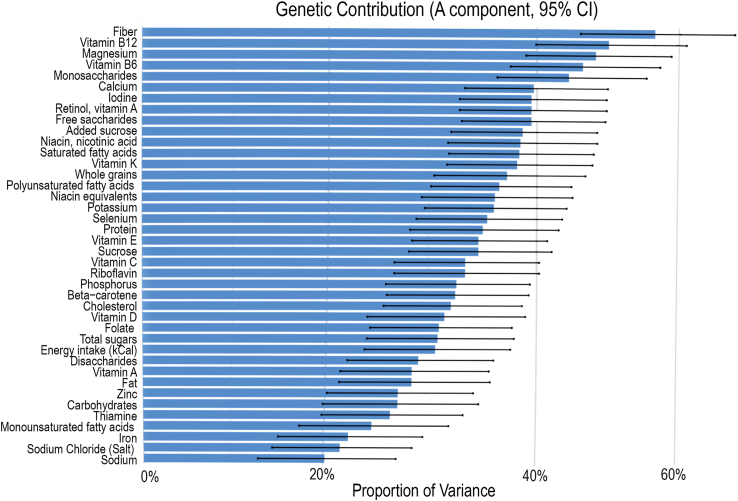
Estimation of the proportion of variance explained by the additive genetic effect for intake of energy (kcal) and nutrients. Error bars display the 95% confidence interval (CI).

Variation in total energy and nutrient intake was analyzed, and the total variance was decomposed into genetic and environmental components using classical twin modeling. The AE model was favored for estimating the genetic contribution for nutrients and energy intake (*n* = 40 variables) (Supplemental Table 6). The genetic influence ranged from 50.0% to 20.2%. For the following nutrients, the variance of intake explained by differences in additive genetic factors exceeded 40%: fiber 50.0% (43.8%, 56.2%), vitamin B12 46.2% (39.9%, 52.5%), magnesium 45.1% (39.0%, 51.3%), vitamin B6 44.0% (37.6%, 50.4%), monosaccharides 42.8% (36.4%, 49.3%). Just <40% the following additive genetic effects were as follows: calcium 39.7% (33.4%, 46.1%), iodine 39.5% (32.9%, 46.0%), retinol 39.5% (32.9%, 46.1%), vitamin A 39.5% (21.5%, 25.8%), free saccharides 39.5% (33.1%, 45.9%), and added sucrose 38.7% (32.1%, 45.2%). No nutrient was identified with a genetic contribution <20% (Figure 4 and Supplemental Table 6). These findings emphasize the importance of genetic factors influencing total energy and nutrient intake patterns.

### Genetic driving forces behind different taste preferences

**FIGURE 5:**
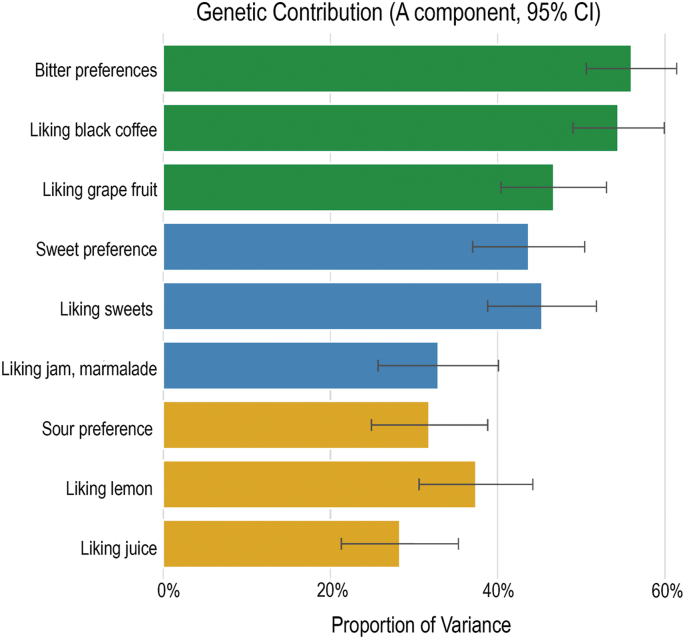
Estimation of the proportion of variance explained by the additive genetic effect for taste preferences. Error bars display the 95% confidence interval (CI).

Taste preferences for bitter, sweet, and sour foods were examined, and the variance in preferences was divided into genetic and environmental contributions using classical twin modeling. The AE model was identified as the most parsimonious model to estimate genetic contribution to food preferences across bitter, sweet, and sour tastes. Foods representing bitter taste exhibited the highest additive genetic influence at 56.0% (50.6%, 61.4%), including black coffee 54.4% (49.0%, 59.9%) and grapefruit 46.7% (40.4%, 53.0%). Sweet taste preferences showed a genetic contribution of 43.7% (37.0%, 50.4%), separated into sweets 45.3% (38.8%, 51.8%) and jam and marmalade 32.9% (25.7%, 40.1%). Sour taste preferences had a lower mean genetic influence of 31.8% (23.9%, 38.8%), with juice on 28.3% (21.3%, 35.3%) and lemon 37.4% (30.6%, 44.2%) (Figure 5, Supplemental Table 6). These findings highlight the substantial role of additive genetic factors in the shaping of individual taste preferences.

## Discussion

This population-based twin study examined genetic and environmental contributions to a broad set of dietary characteristics in young Swedish twins, including food selection, dietary patterns, nutrient intakes, and taste preferences. Across all traits assessed, additive genetic effects accounted for a substantial proportion of the observed variation, ranging from 20% to 61%. Shared environmental effects were consistently negligible, whereas the majority of nongenetic variance was explained by nonshared environmental factors. This pattern highlights the importance of individual lifestyle, personal experiences, and contextual influences on dietary intake in young adulthood. The variation in the magnitude of genetic and environmental influences across dietary traits suggests that dietary intake does not represent a single unified behavior but rather a constellation of related behaviors shaped by distinct interactions between biological predisposition and environmental exposures. Traits with stronger genetic influence likely reflect more stable, biologically anchored tendencies, whereas those influenced primarily by environmental factors may be more sensitive to individual experience.

The heritability estimates reported here fall within the range commonly observed in prior twin studies of dietary behavior (Supplemental Table 1). Thus, like our findings earlier, twin research has demonstrated heritability in the range of 20%–60% for intakes in food groups, and for energy and nutrient intakes in adults, and similar agreements are seen for food liking and composite diet quality indices. Despite the consistent evidence that genetics contributes to dietary behavior in the present and previous studies, the fact that most prior investigations focused on narrow segments of diet, together with differences in target populations and analytic approaches, obstructs cross-study comparisons. As a result, inconsistent heritability estimates across studies may reflect both methodological differences and biological variation related to population genetics or exposure patterns. Together, these limitations underscore the need for broader assessments, such as those undertaken in the present investigation, to contextualize the role of genetic and environmental influences within the complexity of dietary behavior. The present study extends previous work by assessing a broad range of dietary features within the same analytic framework, providing a more integrated understanding of how genetic and environmental factors shape diet in young adulthood, and benefits from a substantially larger sample size that enhances statistical power. Notably, the present finding that preference for bitter and sweet was driven by additive genetic effects is well in line with the well-documented innate preference for sweet flavors [30] and aversion to bitter tastes [31] from infancy and onward. Several single-nucleotide polymorphisms, such as in the taste receptor (TAS) genes encoding taste receptor proteins, have been linked to perception and preference of both sweet and bitter [32,33].

The current study benefited from several methodological strengths. The large, population-based cohort drawn from the STR provides a robust sample of young adults whose dietary habits have transitioned beyond direct parental oversight yet remain in a formative stage of life. The use of a comprehensive and validated food-frequency questionnaire provided high-quality data across a wide array of dietary features. Another strength is the assessment of taste preferences using food-based liking ratings, which have shown construct validity in young Swedish adults through consistent associations with laboratory-based taste tests and reported dietary intake [32]. These complementary measures allow for a more thorough depiction of the mechanisms contributing to dietary variation.

Nevertheless, several limitations warrant consideration. As in all long-term cohort studies, some participant attrition occurred over the follow-up period from 9 to 24 y of age, with overall final response rate reported to be 69% [34]. However, available comparisons indicate that the final sample remains broadly representative of Swedish young adults based on comparisons of BMI and proportions reporting a food allergy or food intolerance in population data at the Public Health Agency of Sweden (https://www.folkhalsomyndigheten.se/the-public-health-agency-of-sweden/) and the Swedish Food Agency (https://www.livsmedelsverket.se/en). Furthermore, self-reported dietary data are inherently susceptible to bias. To reduce the impact of reporting inaccuracies, we excluded food records showing signs of underreporting or unacceptable proportions of missing responses, adjusted intake values for estimated energy intake and body weight when appropriate, and excluded participants with extreme BMI values. Although these steps likely minimized bias, measurement error cannot be completely eliminated.

In addition, the classical twin model carries inherent assumptions. It relies on the premise that MZ and DZ twins experience equally relevant environmental exposures and that genetic and environmental influences act additively and independently. Violations of the equal environment assumption could inflate heritability estimates. However, such biases are likely smaller for young adults, who have spent part of their lives outside the shared household environment and with more autonomy to select their own environments. Gene–environment correlation, if present, could also contribute to upwardly biased heritability estimates. In addition, heritability estimates do not identify specific genetic variants or pathways but instead quantify the proportion of variance attributable to aggregate genetic influences. Because nonshared environmental factors accounted for a large portion of the variance in this study, determinants such as lifestyle patterns, social context, and cultural influences remain essential to understanding dietary behavior. Finally, because heritability estimates are population- and context-specific, the findings may not generalize to populations differing in age, ancestry, cultural circumstances, or dietary environments.

Understanding the determinants of dietary behavior is central to informing public health strategies and clinical interventions. Preferences for energy-dense foods, portion sizes, and specific flavor profiles may interact with genetic predispositions to influence long-term health outcomes. Whether dietary interventions are equally effective across traits with differing heritability remains uncertain. It is plausible that behaviors less strongly influenced by genetic factors may be more responsive to intervention, whereas behaviors shaped by stronger genetic predispositions may require more intensive or personalized strategies. Regardless, young adulthood is a critical period during which long-term dietary habits are established, often without direct influence from familial or household factors. Effective guidance during this life stage may benefit not only individuals but also their future children. Future studies may add knowledge on how the genetic and shared environmental influences on dietary behavior shift across the life span and whether polygenic tools may help improve diet-related risk assessment and intervention strategies.

In summary, this study provides a comprehensive characterization of genetic and environmental influences on dietary behavior in young Swedish adults. Additive genetic factors accounted for a substantial proportion of variation across dietary patterns, foods and food groups, nutrients, energy intake, broader eating patterns, and taste preferences. Shared environmental influences were minimal, whereas nonshared environmental factors played a major role across all traits. The present results, which were broadly consistent with our expectations of moderate genetic influence and minimal shared environmental effects, highlight the need for future work to examine potential sex- and age-related differences in greater detail. The findings offer valuable insights for researchers studying the links between diet and diet-related disease and may support efforts to refine prevention and treatment strategies within precision nutrition and public health.

## Author contributions

The authors’ responsibilities were as follows – IJ, LE: initiated the study, designed the FFQ, and created the scripts for nutrient estimations; LK, IJ: performed other dietary calculations; LK: performed statistical analyses with the support of SH and AE; LK, AE: wrote the first draft; and all authors: reviewed and edited the manuscript and agreed to the published version of the manuscript.

## Data availability statement

Data from STR are available as described at https://ki.se/en/research/swedish-twin-registry-for-researchers. The analytical code used in this study will be provided at a reasonable request.

## Funding

The study was supported by an infrastructure grant from the Swedish Research Council to the STR (grant no 2021-00180). The County Council of Västerbotten supported the study through a TUA (RV 97790) and an ALF (RV 979566) grant. SH receives salary funding from Wellcome (227534/Z/23/Z). None of the funders of this study had a role in the design, data collection, analysis, interpretation or decision to publish.

## Conflict of interest

The authors report no conflicts of interest.
